# Small dense LDL cholesterol is associated with metabolic syndrome traits independently of obesity and inflammation

**DOI:** 10.1186/s12986-019-0334-y

**Published:** 2019-01-21

**Authors:** Jiahua Fan, Yangqing Liu, Songping Yin, Nixuan Chen, Xinxiu Bai, Qiuyi Ke, Jia Shen, Min Xia

**Affiliations:** Guangdong Provincial Key Laboratory of Food, Nutrition and Health; Guangdong Engineering Technology Research Center of Nutrition Translation; Department of Nutrition, School of Public Health, Sun Yat-sen University (Northern Campus), Guangzhou, Guangdong Province, People’s Republic of China

**Keywords:** Metabolic syndrome, Small dense LDL cholesterol, Lipid metabolism

## Abstract

**Background:**

Small dense LDL cholesterol (sdLDL-c) has been established to be highly associated with metabolic disorder. However, the relationship between circulating sdLDL-c and the presence of metabolic syndrome (MetS) has not been fully established.

**Methods:**

A total of 1065 Chinese males (45.07 ± 11.08 years old) without diabetes and general obesity was recruited into a population-based, cross-sectional study. The MetS was defined based on the updated National Cholesterol Education Program/ Adult Treatment Panel III criteria for Asian Americans. Serum sdLDL-c concentration was measured by a homogeneous assay method and its relationship with MetS and its traits was investigated.

**Results:**

Serum sdLDL-c concentrations increased gradually with increasing numbers of MetS components (*p* < 0.001) and the proportion of patients with MetS increased gradually with increasing sdLDL-c levels (*p* for trend< 0.001). For the second, third, and fourth sdLDL-c quartiles versus the first, the OR (95% CI) for MetS were 4.47(2.41,8.28), 5.47(2.97,10.07) and 8.39(4.58,15.38) (*p* < 0.001 for trend) after multivariate adjustment. The stratified analysis conducted according to LDL-c levels showed that the OR between serum sdLDL-c levels and MetS was greater in those LDL-c levels lower than 3.3 mmol/L (OR = 22.97; 95% CI, 7.64–69.09) than in those LDL-c levels higher than 3.3 mmol/L (OR = 17.49; 95% CI, 4.43–68.98). Mediation analysis showed sdLDL-c mediated 38.6% of the association of waist circumference with triglycerides, while the association between sdLDL-c and MetS components did not mediate by hsCRP.

**Conclusions:**

This study found that high sdLDL-c concentrations were associated with the presence of MetS independently of central obesity and inflammation.

**Electronic supplementary material:**

The online version of this article (10.1186/s12986-019-0334-y) contains supplementary material, which is available to authorized users.

## Background

Metabolic syndrome (MetS) represents a group of clinical and laboratory abnormalities (central obesity, hypertension, dyslipidemia and hyperglycemia) that augment the risk of developing atherosclerosis, cardiovascular disease, type 2 diabetes mellitus (T2DM) and all-cause of mortality [1–3]. The prevalence of MetS has increased at an alarming rate worldwide over the last two decades [4–6]. In China, the prevalence of MetS has increased by approximately 20% from 2001 to 2010 (9.8% in 2001 vs 31.0% in 2010) according to the criterion of National Cholesterol Education Program (NCEP)-the Adult Treatment Panel III (ATP III), which translates to an estimated 260 million adults with increased MetS [7–9]. Many studies have shown that MetS was an independent risk factor in the pathogenesis of many chronic diseases such as T2DM, cardiovascular disease (CVD) and so forth [10–12]. Although the exact molecular mechanism of MetS remains elusive, identifying the key markers associated with MetS development and progression is very essential.

Low-density lipoprotein (LDL) is one of the body’s lipoproteins and an important carrier of cholesterol in the circulation. High levels of LDL cholesterol (LDL-c) were associated with a high risk of developing CVD [13]. Over the past two decades, interest has grown in the predictive value of LDL particle size, and the determination of LDL particles has been included into the guidelines of the American Association of Clinical Endocrinologists for prevention of atherosclerosis [14]. Small, dense low-density lipoprotein cholesterol (sdLDL-c), a type of smaller LDL-c, is considered an emerging risk factor for T2DM and CVD. SdLDL-c levels have been found the association with elevated triglyceride (TG) levels and low HDL-c concentrations, constitutes the ‘proatherogenic lipoprotein phenotype’, a common feature of T2DM and MetS [15–17]. The potential mechanism maybe partly because of the lower affinity for the LDL receptor and its multiple atherogenic modifications in blood [18]. And subjects with higher sdLDL-c levels have been shown to be associated with an increased risk factor for cardiovascular disease both in cross-sectional and prospective observational studies [19–22].

Moreover, high circulating sdLDL-c levels were also associated with obesity and systemic inflammation [23, 24]. Obesity, which is usually accompanied by systemic inflammation, has long been regarded as the main origin of MetS. Indeed, dyslipidemia and inflammation, the main and unifying hypothesis to describe the pathophysiology of atherosclerosis, are both associated with sdLDL-c [25–27]. In addition, there is an interaction between dyslipidemia and inflammation, the pro-inflammatory pathway can directly affect lipid metabolism, including elevated level of triglyceride-rich very low-density lipoproteins (VLDL), triglyceride (TG) and free fatty acids (FFA) [28]. At high TG levels status, VLDL particles are initially converted to large LDL particles and subsequently converted to small dense LDL, resulting in an increase of sdLDL-c level in circulation [29]. Inversely, lipids can also directly induce an inflammatory reaction, and cholesterol feeding can promote the inflammatory reaction, which in turn may contribute to the development of MetS [30, 31].

However, studies focus on the relationship between sdLDL-c with MetS and its components is limited. Besides, there were no previous studies conducted mediation analysis to investigate the detail of which factors mediate the associations between central obesity, inflammation, sdLDL-c and Mets in humans. The objective of the present study was to evaluate the association of sdLDL-c concentration with the presence of MetS and its components in 1065 Chinese males without general obesity and diabetes.

## Methods

### Subjects and settings

Study participants were recruited from the locoman health screening in Shaoguan Railway Hospital (Shaoguan City, Guangdong Province, China) from April 2016 to August 2017. There were 1842 males aged 20–70 years participated in this cross-sectional study. For the present study, exclusion criteria were the existence of any infectious or significant hematologic disorders, thyroid dysfunction, severe liver and/or renal insufficiency, malignant tumors and those with missing variables. Subjects were also excluded if they were admitted from the Emergency Room or receiving treatment from the clinic, resulting in a total of 1065 participants without diabetes and general obesity for the final analyses. The study protocol was approved by the Institutional Review Board of the Sun Yat-sen University, conducted according to the principles expressed in the Declaration of Helsinki and written informed consent was obtained from all participants.

### Measurements

Participants were interviewed to collect information on demographic characteristics, behavioral habits, medical history and the use of medication by a standardized questionnaire. Smoking habits and alcohol consumption were classified into three groups: never, past, or current. Current smoker was defined as subject who smoked at least one cigarette per day for more than 6 months. Current drinker was defined as subject who drank any type of alcoholic beverage at least once a week for more than half a year. Physical activity defined as taking physical activity at least once a week during the past 12 months.

Clinical measurements of each subject were extracted from an electronic medical record system. Trained nurses measured height, weight, waist circumference and blood pressure using a standard protocol [25]. Body mass index (BMI) was defined as the weight in kilograms divided by the square of height in meters, BMI ≥28 kg/m^2^ was defined as general obesity [32–34]. Men with waist ≥90 cm was defined as central obesity. Blood samples were taken after at least 12-h’s fast in the morning and collected for the measurement of sdLDL-c, which were measured by a homogeneous assay method (sdLDL-EX “Seiken”, Denka Seiken, Tokyo, Japan) using the Hitachi Automatic Analyzer 7600–020 (Hitachi, Tokyo, Japan) [19, 35]. Total blood cholesterol, HDL-c, LDL-c and TG were measured enzymatically using an automatic analyzer (Hitachi 747 autoanalyzer, Hitachi) using a commercial assay kit (Wako Pure Chemical Industries, Osaka, Japan). LDL-c ≥ 3.3 mmol/L (130 mg/dL) was defined as high LDL-c [36]. Serum levels of high-sensitivity C reactive protein (hsCRP) were determined by a high-sensitivity turbidimetric assay (Roche, Basel, Switzerland).

### Definition of MetS

MetS was defined based on the updated National Cholesterol Education Program/ Adult Treatment Panel III criteria for Asian Americans as having at least three of the following components: 1) waist circumference ≥ 90 cm for men or ≥ 80 cm for women; 2) TG ≥1.7 mmol/L; 3) HDL cholesterol <1.03 mmol/L for men or <1.30 mmol/L for women; 4) blood pressure (BP) ≥130/85 mmHg or current use of antihypertensive medications; 5) fasting plasma glucose (FPG) ≥5.6 mmol/L, previously diagnosed type 2 diabetes or treatment with oral antidiabetic agents or insulin [37] .

### Statistical analysis

Data were expressed as the mean ± standard deviation (SD) for normally distributed variables, whereas median with interquartile range (IQR) for variables with a skewed distribution and numbers (percentages) for the categorical variables. Student’s T-test, ANOVA, Mann-Whitney U test or Kruskal-Wallis test was used for the comparisons between continuous variables. Differences between categorical data were assessed with the χ2 test. Adjusted mean differences in metabolic variables across sdLDL-c quartiles were calculated using linear regression analysis. Odds Ratio (OR) and 95% confidence interval (95% CI) was performed by binary Logistic regression analysis to quantify the relationship between sdLDL-c with MetS and its clusters. Stratified analysis was used to evaluate the effect of LDL-c level on the association between sdLDL-c and MetS. A causal mediation analysis, the Process Macro for SPSS, was used to analyze the extent to which sdLDL-c explains the association of central obesity with MetS components and the extent to which hsCRP mediates the effect of sdLDL-c on MetS component values. Effects were estimated by means of nonparametric bootstrapping with 5000 resamples and percentile-based confidence intervals [38]. A two-sided *P* < 0.05 were considered statistically significant. Statistical analyses were performed using the SPSS program (ver 20.0, SPSS).

## Results

### Characteristics of study participants

**Fig. 1:**
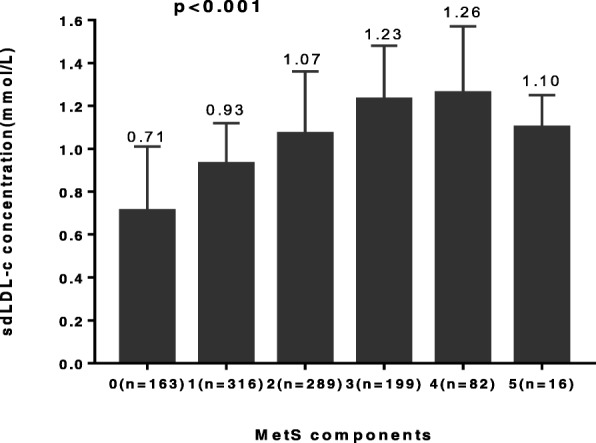
Serum sdLDL-c concentration according to the number of MetS components

The baseline demographic and clinical characteristics of the study participants are summarized in Table 1. The average age was 45.07 ± 11.08 years of all the subjects, of whom 27.9% had MetS and 31.3% were central obesity. The median sdLDL-c concentration was 1.03(0.73,1.31) mmol/L and the mean hsCRP concentration was 1.78 ± 3.28 mg/L. Compared to subjects without MetS, subjects with MetS had higher sdLDL-c and hsCRP levels (both *p* < 0.05). In addition, the sdLDL-c/LDL-c was significantly higher in MetS subjects compared with non-MetS subjects (*p* < 0.001), while levels of LDL-c were similar in subjects with or without MetS (*p* > 0.05). SdLDL-c level increased gradually with the number of MetS components (Fig. 1). The sdLDL-c levels increased gradually from the subjects without MetS (0.71 mmol/L) to those with all 5 MetS components (1.10 mmol/L).

**Table 1:** Baseline characteristics of subjects with or without the MetS

Characteristics	Total (*N* = 1065)	Metabolic syndrome (*n* = 297)	Non-metabolic syndrome (*n* = 768)	*P* value
Age, years	45.07 ± 11.08	48.57 ± 8.55	43.71 ± 11.64	< 0.001
Current Smoker, n (%)	643 (60.4)	185 (62.6)	457 (59.5)	0.225
Current Drinker, n (%)	423 (39.7)	115 (40.1)	308 (40.1)	0.642
Physical activity, n (%)	758 (71.2)	208 (70.0)	550 (71.6)	0.610
Hypertension, n (%)	96 (9.0)	45 (15.2)	51 (6.6)	< 0.001
Central obesity, n (%)	333 (31.3)	202 (68.0)	131 (17.1)	< 0.001
BMI, kg/m^2^	23.53 ± 2.46	24.94 ± 2.03	22.98 ± 2.4	< 0.001
Waist circumference, cm	85.11 ± 7.88	90.41 ± 6.54	83.06 ± 7.39	< 0.001
TC, mmol/L	5.44 ± 1.12	5.61 ± 1.10	5.37 ± 1.12	0.002
TG, mmol/L	1.94 ± 1.37	2.92 ± 1.74	1.56 ± 0.96	< 0.001
HDL-c, mmol/L	1.25 ± 0.28	1.11 ± 0.27	1.31 ± 0.26	< 0.001
LDL-c, mmol/L	3.56 ± 0.93	3.52 ± 0.98	3.58 ± 0.90	0.290
SBP, mm Hg	127.82 ± 15.04	134.52 ± 14.54	125.23 ± 14.43	< 0.001
DBP, mm Hg	87.26 ± 11.14	92.42 ± 10.08	85.26 ± 10.89	< 0.001
FPG, mmol/L	5.19 ± 0.57	5.47 ± 0.61	5.08 ± 0.51	< 0.001
hsCRP, mg/L	1.78 ± 3.28	2.25 ± 3.16	1.60 ± 3.31	0.004
SdLDL-c/LDL-c, %	28.31 (22.03,35.32)	34.79 (29.63,41.15)	25.91 (20.64,32.11)	< 0.001
sdLDL-c, mmol/L	1.03 (0.73,1.31)	1.23 (0.95,1.50)	0.94 (0.66,1.22)	< 0.001

Values were means±SD or medians in cases of continuous variables and numbers (percentages) in case of categorical data. For differences across the groups of calculated with χ2 test and analysis of variance for categorical and continuous data, respectively. *sdLDL-c* small dense low-density lipoprotein-cholesterol, *BMI* body mass index, *HDL* high-density lipoprotein, *SBP* systolic blood pressure, *DBP* diastolic blood pressure, *TC* total cholesterol, *TG* triglyceride, *HDL-c* high-density lipoprotein-cholesterol, *LDL-c* low-density lipoprotein-cholesterol, *hsCRP* high-sensitivity C-reactive protein, *FPG* fasting plasma glucose, *n* number. To convert sdLDL-c to mg/dL, multiply by 38.6

### Correlations between serum sdLDL-c levels and MetS–related parameters

Partial Spearman correlation analysis showed that sdLDL-c was significantly positively correlated with MetS–related parameters, such as TG, HDL-c, FPG and waist circumference after adjusting for age, smoking, drinking and physical activity (*p* < 0.001, Table 2). The strongest correlation was between sdLDL-c and TG (r = 0.454, *p* < 0.001). Besides, the sdLDL-c level was significantly positively correlated with LDL-c (r = 0.609, *p* < 0.001), while there was no significant correlation between sdLDL-c and hsCRP (r = 0.051, *p* = 0.100).

**Table 2:** Multivariable-adjusted Spearman correlation coefficients of serum sdLDL-c concentration and MetS-related parameters

	r	*P*
TG	0.454	< 0.001
HDL	0.145	< 0.001
FPG	0.116	< 0.001
SBP	0.018	0.568
DBP	0.026	0.393
Waist circumference	0.134	< 0.001
LDL	0.609	< 0.001
hsCRP	0.051	0.100

Spearman correlation coefficients were adjusted by age, smoking, drinking and physical activity; *sdLDL-c* Small dense LDL cholesterol, *MetS* Metabolic syndrome, *TG* Triglyceride, *HDL* high-density lipoprotein, *LDL* Low-density lipoprotein cholesterol, *hsCRP* high-sensitivity C reactive protein, *FPG* fasting plasma glucose, *BP* Blood pressure;

### Association of serum sdLDL-c levels with MetS and its components

The proportion of MetS and its related metabolic disorders increased across sdLDL-c quartiles. Multiple logistic regression analysis showed that the sdLDL-c level was associated with high waist circumference, high TG and low HDL-c, independent of age, smoking, drinking and physical activity (Table 3, model 1). After additionally adjusting for FPG, hsCRP and anthropometric measurements, the association remained significantly between sdLDL-c and MetS-related dyslipidemia (high TG and low HDL-c), and high TG concentration had the strongest association with sdLDL-c level (Table 3, model 2). Subjects with higher quartiles of sdLDL-c had an increased likelihood of having MetS compared with those in the lowest quartile. The ORs (95% CI) for MetS in the second, third, and fourth sdLDL-c quartile versus the first were 4.47(2.41,8.28), 5.47(2.97,10.07) and 8.39(4.58,15.38), respectively (*p* < 0.001 for trend, Table 3, model 2).

**Table 3:** Percentages and adjusted ORs (95% CI) for MetS and its related components for comparison of the three highest sdLDL-c quartiles with the first quartile

Variables	Quartile of serum sdLDL-c, mmol/L				*P* for trend
	Q1 (< 0.73) *n* = 265	Q2 (0.73–1.03) *n* = 267	Q3 (1.03–1.31) n = 267	Q4 (≥1.31) *n* = 266	
High waist circumference (%)	20.5	30.0	34.1	40.6	< 0.001
Model 1	reference	1.44 (0.96,2.16)	1.59 (1.06,2.38)	2.05 (1.37,3.06)	0.006
Model 2^a^	reference	1.32 (0.79,2.22)	1.46 (0.88,2.44)	1.67 (1.00,2.77)	0.254
High TG (%)	4.2	32.6	58.4	78.2	< 0.001
Model 1	reference	11.16 (5.77,21.61)	32.97 (17.01,63.88)	85.09 (42.97,168.48)	< 0.001
Model 2	reference	8.91 (4.88,16.24)	27.30 (14.93,49.93)	65.93 (35.26,123.29)	< 0.001
Low HDL-c (%)	14.8	23.6	24.0	15.8	0.006
Model 1	reference	1.85 (1.18,2.90)	1.88 (1.19,2.97)	1.13 (0.69,1.84)	0.006
Model 2	reference	1.70 (1.10,2.64)	1.520.96,2.38)	1.03 (0.64,1.65)	0.027
High BP (%)	51.1	64.8	62.9	67.7	0.001
Model 1	reference	1.54 (1.08,2.21)	1.29 (0.90,1.85)	1.57 (1.08,2.27)	0.055
Model 2	reference	1.49 (1.03,2.17)	1.48 (1.03,2.13)	1.15 (0.79,1.67)	0.158
High FPG (%)	17.0	19.5	25.8	26.7	0.016
Model 1	reference	1.03 (0.65,1.61)	1.40 (0.90,2.16)	1.41 (0.90,2.15)	0.235
Model 2^a^	reference	1.07 (0.69,1.66)	1.32 (0.86,2.02)	1.34 (0.87,2.06)	0.434
High hsCRP (%)	9.1	10.5	16.5	13.9	0.043
Model 1	reference	1.05 (0.59,1.88)	1.69 (0.98,2.91)	1.69 (0.98,2.91)	0.171
Model 2^a^	reference	1.10 (0.62,1.93)	1.62 (0.93,2.75)	1.44 (0.83,2.49)	0.259
Metabolic syndrome (%)	7.2	25.8	34.1	44.0	< 0.001
Model 1	reference	4.06 (2.35,7.01)	5.56 (3.24,9.52)	8.46 (4.96,14.44)	< 0.001
Model 2	reference	4.47 (2.41,8.28)	5.47 (2.97,10.07)	8.39 (4.58,15.38)	< 0.001

ORs were estimated from Logistic regression models; Model 1 was adjustment for age, smoking, drinking and physical activity; Model 2 was additionally adjustment for FPG, hsCRP, BMI, and waist circumference based on model 1; Percentages shown in the table were unadjusted results; ^a^The variable used in the direct definition of the outcome variable (FPG, waist circumference or hsCRP) was excluded from the adjustment variables

### The effect of LDL-c level on the association between sdLDL-c and MetS

**Fig. 2:**
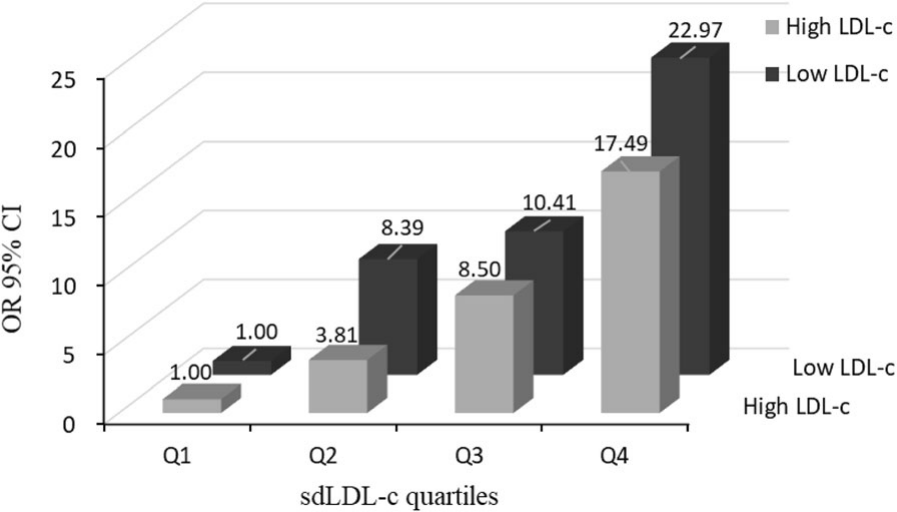
Effect of LDL-c subgroups on the association between sdLDL-c and MetS

Stratified analysis of the effect of LDL-c level on the association between sdLDL-c and MetS. Results showed that the OR (95% CI) between serum sdLDL-c levels and MetS was 22.97(7.64,69.09) in group of those LDL-c levels lower than 3.3 mmol/L, whereas in the group of those LDL-c level higher than 3.3 mmol/L, the OR (95% CI) was 17.49(4.43,68.98), which indicates the association was more prominent in the group of those LDL-c levels lower than 3.3 mmol/L (Fig. 2). Furthermore, results showed that the proportion of patients with MetS increased gradually with increasing sdLDL-c/LDL-c level (*p* for trend< 0.001) and the OR between sdLDL-c/LDL-c level and MetS was significantly elevated in the higher sdLDL-c/LDL-c quartiles. (Additional file 1: Fig. S1 and Table S1).

### SdLDL-c mediated the effect of waist circumference on MetS–related parameters

Causal mediation analysis showed that sdLDL-c mediated 38.6% of the association between waist circumference with TG concentration and 13.5% of the association of waist circumference with FPG (Table 4). FPG mediated about 7.3% of the association of sdLDL-c with BMI (Additional file 1: Table S2). While hsCRP did not mediate any of the associations of sdLDL-c and MetS (Additional file 1: Table S3). This result was consistent with the finding in Table 2 that there was no association between sdLDL-c and hsCRP.

**Table 4:** SdLDL-c mediated fraction of the effect of waist circumference on MetS–related parameters

Outcome	Total	Direct	Mediated	Mediated%
BMI, kg/m^2^	0.2495 (0.2375,0.2615)	0.2488 (0.2367,0.2610)	0.0007 (−0.0009,0.0025)	–
*P* value	< 0.001	< 0.001	> 0.05	–
TG, mmol/L	0.0277 (0.0170,0.0385)	0.0171 (0.0073,0.0268)	0.0107 (0.0059,0.0156)	38.63 (21.30,56.32)
*P* value	< 0.001	< 0.001	< 0.05	< 0.05
HDL, mmol/L	−0.0054 (−0.0075,-0.0032)	− 0.0062 (− 0.0084,-0.0040)	0.0008 (0.0004,0.0014)	–
*P* value	< 0.001	< 0.001	< 0.05	–
SBP, mmHg	0.3605 (0.2452,0.4759)	0.3625 (0.2461,0.4789)	−0.0020 (− 0.0178,0.0140)	–
*P* value	< 0.001	< 0.001	> 0.05	–
DBP, mmHg	0.2186 (0.1325,0.3047)	0.2174 (0.1305,0.3044)	0.0012 (−0.0105,0.0141)	–
*P* value	< 0.001	< 0.001	> 0.05	–
FPG, mmol/L	0.0074 (0.0029,0.0118)	0.0063 (0.0019,0.0108)	0.0010 (0.0004,0.0019)	13.51 (5.41,25.68)
*P* value	0.001	0.005	< 0.05	< 0.05
hsCRP, mg/L	0.0382 (0.0121,0.0642)	0.0359 (0.0096,0.0621)	0.0023 (−0.0016,0.0068)	–
*P* value	0.004	0.008	> 0.05	–

The total effect, direct effect, and sdLDL-c mediated effect of waist circumference on each MetS-related parameter and the proportion of the total effect of waist circumference that is mediated by sdLDL-c were showed in the table; The 95% CI was calculated by nonparametric bootstrapping, Process Macro for SPSS; The model was adjusted for age, smoking, drinking and physical activity

## Discussion

Small dense LDL cholesterol (sdLDL-c), is a distinct LDL cholesterol subclass, which is associated with raised TG and decreased HDL-c levels in adiposity and diabetes, playing a distinct metabolic role in atherosclerosis [2–4]. However, the relationship of sdLDL-c with MetS and its components has not been fully established. In the present study, we found that sdLDL-c was strongly associated with MetS and its components, independently of central obesity and inflammation in subjects without general obesity and diabetes. Our results showed that sdLDL-c level increased gradually with the number of MetS components and the proportion of patients with MetS increased gradually with increasing sdLDL-c levels. Besides, the OR between serum sdLDL-c levels and MetS was greater in lower LDL-c levels than in higher LDL-c levels, suggesting that the association between sdLDL-c and MetS was independent of LDL-c levels. Notably, the causal mediation analysis revealed that sdLDL-c mediated 38.6% of the association between waist circumference and TG concentration, while hsCRP did not mediate any of the associations of sdLDL-c and MetS. Thus, our findings indicated that a higher level of sdLDL-c might be a key marker associated with MetS progression even before the appearance of central obesity, diabetes and inflammation.

MetS is a complex of clinical features and the main feature of which is obesity. Individuals with obesity show characteristic imbalance of metabolic profile which is associated with profound changes in insulin sensitivity, inflammatory reaction and other biochemical metabolites alterations, making an individual more potential to metabolic disorders [39–42]. Studies have found an increased level of plasm sdLDL-c particles in general obese participants [43]. Our study also found a positive association of sdLDL-c with FPG and waist circumference, a simple anthropometric measure of central obesity, after multivariate adjustment. However, due to the lack of evidence for the detail of which factors mediate the associations between central obesity, diabetes and sdLDL-c in humans, determining which of their pathway associated with MetS acts first is difficult. Mediation analysis demonstrated that sdLDL-c only explained 13.5% of the waist circumference with FPG and FPG did not mediate any of the association of sdLDL-c with MetS, except 7% of the association of sdLDL-c with BMI. Besides, the present study including subjects without general obesity and diabetes, we were able to address the association between sdLDL-c and MetS was independent of waist circumference and FPG, indicating that central obesity may not be an original cause of the relationship between sdLDL-c level and MetS.

The inflammatory reaction is also an important predictor of MetS and usually accompanied with obesity, which is usually performed as a low grade, systemic inflammation, such as C-reactive protein [44, 45]. Study has found that higher hsCRP levels correlated with smaller LDL size and implied that low-grade inflammation was closely linked to alterations in lipoprotein metabolism [46]. In our study, however, we found there was no significant relationship between sdLDL-c and hsCRP after adjusting for other variables. The reason for the inconsistent results compared with former studies were likely due to the subjects in our study are non-obese and non-diabetes, both are the main causes of inflammation [40]. Furthermore, studies found that increased oxidative stress played an important role in the initiation and progression of metabolic disorders [47, 48]. SdLDL-c is the precursor of oxidized LDL (ox-LDL) and study has revealed that higher ox-LDL concentrations were associated with MetS and its components independently of central obesity and insulin resistance [49], suggesting that oxidative stress may represent the mechanistic pathway through which high sdLDL-c status promote the metabolic disorders. Accordingly, our study indicated that sdLDL-c is directly associated with MetS and its components, initially acting in parallel with central obesity and inflammation.

A possible explanation for the result that sdLDL-c was strongly associated with MetS and its components, independent of LDL-c, central obesity, inflammation and other variables, is the shift in the metabolites used to produce energy [50]. Energy metabolism disorder is the main reason, while dyslipidemia, central obesity, inflammation, oxidative stress and diabetes emerging as secondary consequences. Normally, the apo B-containing triglyceride-rich VLDL, which is secreted by the liver, was under the action of hepatic lipase to form normal-size LDL that remains in circulation for 2 days. At high TG condition, VLDL particles are initially converted to large LDL particles then thereafter converted to sdLDL-c, which can remain in circulation for about 5 days [29]. Our study showed that sdLDL-c mediated 38.6% of the association between waist circumference and TG concentration, which was the strongest that we found among the MetS components, maybe partly because of their common participation in lipids pathways. In addition, the composition of HDL-c can be altered in the high VLDL condition, leading to increased catabolism of lipid particles [51]. We found that as the increase of sdLDL-c level, HDL-c level increased significantly in the fourth quartile of sdLDL-c levels. This result is consistent with the recent study that a U-shaped association between HDL-c and mortality was found, suggesting that an extremely high concentration of HDL-c is a risk factor for metabolic disorders [52, 53]. Maybe the potential trigger for elevated HDL-c levels associated with high mortality is due to the high level of sdLDL-c, but the mechanisms require investigating in the future study.

There were several limitations to our study. Firstly, this was a cross-sectional study, which is unable to identify any causal relationships between sdLDL-c and MetS. Secondly, regressions were adjusted for the main potential confounders, but there may still exist some residual confounding because of unmeasured or unknown confounders. Third, we do not detect the level of HOMA-IR, which is the marker of insulin resistance. Finally, our study includes only middle-aged Chinese males, whether such mediation effect was present in females, other populations or other age groups should be studied in the future.

## Conclusion

In conclusion, this study shows that higher sdLDL-c concentrations are associated with MetS and its components independently of central obesity and inflammation. What’s more, the association between sdLDL-c and MetS is more pronounced in lower LDL-c levels than in higher LDL-c levels. Levels of sdLDL-c may play a role as the key marker associated with MetS and its components in parallel with central obesity and inflammation.

## Additional file

Additional file 1: **Figure S1.** The relationship between sdLDL-c/LDL-c ratio and prevalence of MetS. **Table S1.** Logistic regression analysis of the relationship between sdLDL-c/LDL-c ratio and MetS. **Table S2.** FPG mediated fraction of the effect of sdLDL-c on MetS–related parameters. **Table S3.** hsCRP–mediated fraction of the effect of sdLDL-c on metabolic syndrome–related. (DOC 92 kb)

## Abbreviations

95% CI: 95% confidence interval
ATP III: Adult Treatment Panel III
BMI: Body mass index
BP: Blood pressure
CVD: Cardiovascular disease
FPG: fasting plasma glucose
HDL: high-density lipoprotein
hsCRP: high-sensitivity C reactive protein
IQR: Interquartile range
LDL: Low-density lipoprotein
MetS: Metabolic syndrome
NCEP: National Cholesterol Education Program
OR: Odds Ratio
ox-LDL: oxidized LDL
SD: Standard deviation
sdLDL-c: Small dense LDL cholesterol
T2DM: type 2 diabetes mellitus
TC: total cholesterol
TG: Triglyceride
VLDL: Very low-density lipoproteins
