# Supplementary material for: Small dense LDL cholesterol is associated with metabolic syndrome traits independently of obesity and inflammation
Source: Nutr Metab (Lond). 2019 Jan 21;16:7. doi: 10.1186/s12986-019-0334-y (PMC6341753; doi:10.1186/s12986-019-0334-y)
Supplement: Supplementary file 1 — Figure S1. The relationship between sdLDL-c/LDL-c ratio and prevalence of MetS. Table S1. Logistic regression analysis of the relationship between sdLDL-c/LDL-c ratio and MetS. Table S2. FPG mediated fraction of the effect of sdLDL-c on MetS–related parameters. Table S3. hsCRP–mediated fraction of the effect of sdLDL-c on metabolic syndrome–related. (DOC 92 kb) [file 12986_2019_334_MOESM1_ESM.doc]

**Additional file1:**

**Small dense LDL cholesterol is associated with metabolic syndrome traits independently of obesity and inflammation**

Abbreviated title: Small dense LDL and metabolic syndrome

Jiahua Fan^1*^, Yangqing Liu^1*^, Songping Yin^1^, Nixuan Chen^1^, Xinxiu Bai^1^, Qiuyi Ke^1^, Jia Shen^1^, Min Xia^1^

^1^ Guangdong Provincial Key Laboratory of Food, Nutrition and Health; Guangdong Engineering Technology Research Center of Nutrition Translation; Department of Nutrition, School of Public Health, Sun Yat-sen University (Northern Campus), Guangzhou, Guangdong Province, P.R. China.

* These Authors contributed equally to the work.

Address all correspondence and requests for reprints to: Min Xia, Department of Nutrition, School of Public Health, Sun Yat-sen University (Northern Campus), Guangzhou, Guangdong Province, China. E-mail: xiamin@mail.sysu.edu.cn


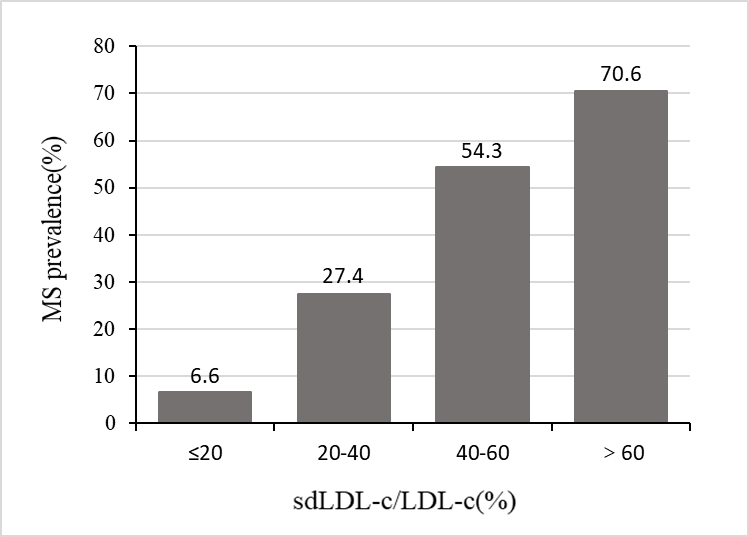


Fig S1 The relationship between sdLDL-c/LDL-c ratio and prevalence of MetS

Table S1 Logistic regression analysis of the relationship between sdLDL-c/LDL-c quartiles and MetS

| Model | sdLDL-c/LDL-c quartiles, % | | | | P for trend |
| --- | --- | --- | --- | --- | --- |
|  | Q1(<22.03) | Q2(22.03-28.30) | Q3(28.31-35.31) | Q4(≥35.32) |  |
| Unadjusted | reference | 3.05(1.67,5.48) | 8.02(4.62,13.93) | 15.85(9.17,27.40) | <0.001 |
| Adjusted | reference | 2.80(1.55,5.06) | 7.24(4.14,12.67) | 14.31(8.21,24.95) | <0.001 |

Adjusted model was adjustment for age, smoking, drinking and physical activity.

Table S2 FPG mediated fraction of the effect of sdLDL-c on MetS–related parameters

| **Outcome** | **Total** | **Direct** | **Mediated** | **Mediated%** |
| --- | --- | --- | --- | --- |
| Waist Circumference, cm | 2.3718(1.3164,3.4272) | 2.1975(1.1383,3.2566) | 0.1743(0.0413,0.3474) | 7.34(1.74,14.64) |
| *P* value | <0.001 | <0.001 | P<0.05 | P<0.05 |
| TG, mmol/L | 1.4436(1.2727,1.6144) | 1.4491(1.2770,1.6212) | -0.0056(-0.0308,0.0176) | - |
| *P* value | <0.001 | <0.001 | p>0.05 | - |
| HDL, mmol/L | 0.0936(0.0550,0.1323) | 0.0921(0.0532,0.1310) | 0.0015(-0.0032,0.0067) | - |
| *P* value | <0.001 | <0.001 | p>0.05 | - |
| SBP, mmHg | 0.6022(-1.4692,2.6736) | -0.0574(-2.1153,2.0004) | 0.6596(0.2770,1.1329) | 109.53(45.99,188.13) |
| *P* value | 0569 | 0.956 | P<0.05 | P<0.05 |
| DBP, mmHg | 0.6691(-0.8681,2.2063) | 0.2709(-1.2634,1.8053) | 0.3982(0.1546,0.7051) | 59.51(23.11,105.38) |
| *P* value | 0.393 | 0.729 | P<0.05 | P<0.05 |
| hsCRP, mg/L | 0.3875(-0.0737,0.8486) | 0.3728(-0.0916,0.8373) | 0.0146(-0.0377,0.0746) | - |
| *P* value | 0.099 | 0.116 | p>0.05 | - |

The total effect, direct effect, and FPG mediated effect of sdLDL-c on each MetS-related parameter and the proportion of the total effect of sdLDL-c that was mediated by FPG were showed in the table; The 95% CI was calculated by nonparametric bootstrapping, Process Macro for SPSS; The model was adjustment for age, smoking, drinking and physical activity. Results in gray indicates maybe the existence of suppression phenomenon.

Table S3 hsCRP–mediated fraction of the effect of sdLDL-c on metabolic syndrome–related parameters

| **Outcome** | **Total** | **Direct** | **Mediated** | **Mediated%** |
| --- | --- | --- | --- | --- |
| Waist Circumference, cm | 2.3718(1.3164,3.4272) | 2.2991(1.2453,3.3528) | 0.0727(-0.0168,0.2253) | - |
| *P* value | <0.001 | <0.001 | p>0.05 | - |
| TG, mmol/L | 1.4436(1.2727,1.6144) | 1.4375(1.2648,1.6065) | 0.0079(-0.0013,0.0333) | - |
| *P* value | <0.001 | <0.001 | p>0.05 | - |
| HDL, mmol/L | 0.0936(0.0550,0.3366) | 0.0967(0.0582,0.1352) | -0.0031(-0.099,0.0008) | - |
| *P* value | <0.001 | <0.001 | p>0.05 | - |
| SBP, mmHg | 0.6022(-1.4692,2.2736) | 0.6240(-1.4509,2.6989) | -0.0218(-0.1474,0.1057) | - |
| *P* value | 0.569 | 0.555 | p>0.05 | - |
| DBP, mmHg | 0.6691(-0.8681,2.2063) | 0.6570(-0.8828,2.1968) | 0.0121(-0.0576,0.1435) | - |
| *P* value | 0.393 | 0.403 | p>0.05 | - |
| FPG, mmol/L | 0.1507(0.0727,0.2287) | 0.1496(0.0715,0.2277) | 0.0011(-0.0034,0.0080) | - |
| *P* value | <0.001 | <0.001 | p>0.05 | - |

The total effect, direct effect, and hsCRP mediated effect of sdLDL-c on each MetS-related parameter and the proportion of the total effect of sdLDL-c that is mediated by hsCRP were showed in the table; The 95% CI was calculated by nonparametric bootstrapping, Process Macro for SPSS; The model was adjustment for age, smoking, drinking and physical activity.
